# Monument in Memory of Dr. Horace Wells

**Published:** 1875-06

**Authors:** 


### ARTICLE IX.

### *Monument in Memory of Dr. Horace Wells.*

Nearly a quarter of a century has passed since Horace Wells, the discoverer of Anæsthesia,—a safe, speedy, and effectual means of abolishing sensibility and consciousness, died.

No monument has yet been erected to perpetuate the memory of Dr. Wells, or, in connection with his name, to commemorate this wonderful discovery. He gave most willingly and cheerfully, wishing it, in his own words, to be "free as air," the use of this boon to humanity; asking of his fellow-men, in return, nothing beyond the proper appreciation of its worth, and the honor that justly belonged to the discoverer. As its importance became more widely known, and the world learned by experience the amazing value of the discovery, the feeling was naturally awakened, that some positive movement should be made towards the accomplishment of this long-delayed duty.

Entertaining this sentiment, doubtless, the Legislature of Connecticut, some two years ago, appropriated Five Thousand Dollars ($5,000) for this purpose, and the city of Hartford a like sum; and under the direction of a Committee a colossal statue in bronze of Dr. Wells has been executed by Truman H. Bartlett, Esq., and will soon be ready for erection on some commanding site in the beautiful Park in the city of Hartford, where the discoverer lived, where the grand idea which was to embalm his name and memory in the hearts of his fellow men everywhere, had its birth, and where his remains now rest.

It is upon the Pedestal, which should be also of bronze, and its ornamentation, that any further funds will need to be expended. This will admit of high and costly adornment in bas-reliefs, in inscriptions, etc., suited to exemplify the uses of the discovery, at the same time it commemorates the discoverer; and we are informed by the most competent judges, will admit of large outlay without transcending the limits of a severe and correct taste.

In view of this circumstance, and of the fact, also, that as the subject has been more freely canvassed, an earnest desire has been expressed in many quarters, both in and out of the State, to take part in this undertaking, it has been thought to be expedient, for the purpose of gratifying this wish, and in order to make the work itself more nearly represent the character and value of the service rendered to mankind by Dr. Wells, to receive such subscriptions from Physicians and Dentists abroad, and through their agency from the public, in the various parts of the country, as they may feel disposed to make. Our appeal is made primarily to the Medical Faculty and Dental Profession, not so much because they have a higher personal interest in the subject than others, but because they, of all men, best know the inestimable value of this discovery to the race.

The Committee who submit the foregoing, represent the Medical and Dental Societies of Hartford, and, in so far as our object shall meet the views of our brethren elsewhere, we respectfully ask from them such friendly aid pecuniarily, as they may think proper to give us, and especially that they take such measures to bring the subject to the notice of their friends and the public as, in their wisdom, they shall consider most likely to receive a favorable response.

Letters of inquiry may be addressed to Dr. E. K. Hunt, Chairman of the Committee of the Hartford Medical Society. Subscriptions may be forwarded to Dr. G. W. Russell, Treasurer, Hartford, Conn.

E. K. Hunt, M. D.,<br>

M. Storrs, M. D.<br>

Jas. Campbell, Jr., M. D } Committee of the Hartford Medical Society.<br>

Dr. Jas. McManus, Dental Committee.

Hartford, Conn., Feb. 15th, 1875.

We approve the object of the Circular, and commend it to the attention of the Medical Profession.

Frs. Bacon, M. D., New Haven.
D. L. Daggett, M. D., New Haven.
C. A. Lindsley, M. D., New Haven.
Dr. H. J. Stevens, Dentist, New Haven.
Gideon L. Platt, M. D., Waterbury.
Geo. H. Waters, Dentist, Waterbury.

New Haven, March 15th, 1875.

---

I approve the object most heartily, but my best endorsement is the enclosed check for fifty dollars. Wishing your and our enterprise every success, etc.

New York, March, 1875. J. Marion Sims.

---

The undersigned cheerfully concur in the effort that is being made to commemorate the name of Dr. Wells, in connection with the discovery of the use of Nitrous Oxide Gas as an agent for producing insensibility during surgical operations.

Gerdon Buck, M. D.
A. L. Northrop, D. D. S., 44 W. 46th St., No. 7.
Chas. E. Francis, D. D. S., 33 W. 47th St.

New York, April 13th, 1875.

---

Colton Dental Association, Cooper Institute,
New York, March 27th, 1875.

T. H. Bartlett—

Dear Sir:—I have received the Wells Circular—or, rather, a proposed copy—and heartily approve of it. I shall this day send my check for fifty dollars to the Treasurer, Dr. G. W. Russell.

Respectfully, yours,
G. Q. Colton.

